# A latent pool of neurons silenced by sensory-evoked inhibition can be recruited to enhance perception

**DOI:** 10.1016/j.neuron.2024.04.015

**Published:** 2024-05-09

**Authors:** Oliver M. Gauld, Adam M. Packer, Lloyd E. Russell, Henry W.P. Dalgleish, Maya Iuga, Francisco Sacadura, Arnd Roth, Beverley A. Clark, Michael Häusser

**Affiliations:** 1 Wolfson Institute for Biomedical Research, University College London, London WC1E 6BT, UK; 2 Sainsbury Wellcome Centre, University College London, London W1T 4JG, UK; 3 Department of Physiology, Anatomy and Genetics, University of Oxford, Oxford OX1 3PT, UK

## Abstract

To investigate which activity patterns in sensory cortex are relevant for perceptual decision-making, we combined two-photon calcium imaging and targeted two-photon optogenetics to interrogate barrel cortex activity during perceptual discrimination. We trained mice to discriminate bilateral whisker deflections and report decisions by licking left or right. Two-photon calcium imaging revealed sparse coding of contralateral and ipsilateral whisker input in layer 2/3, with most neurons remaining silent during the task. Activating pyramidal neurons using two-photon holographic photostimulation evoked a perceptual bias that scaled with the number of neurons photostimulated. This effect was dominated by optogenetic activation of non-coding neurons, which did not show sensory or motor-related activity during task performance. Photostimulation also revealed potent recruitment of cortical inhibition during sensory processing, which strongly and preferentially suppressed non-coding neurons. Our results suggest that a pool of non-coding neurons, selectively suppressed by network inhibition during sensory processing, can be recruited to enhance perception.

## Graphical abstract

**Graphical abstract:**
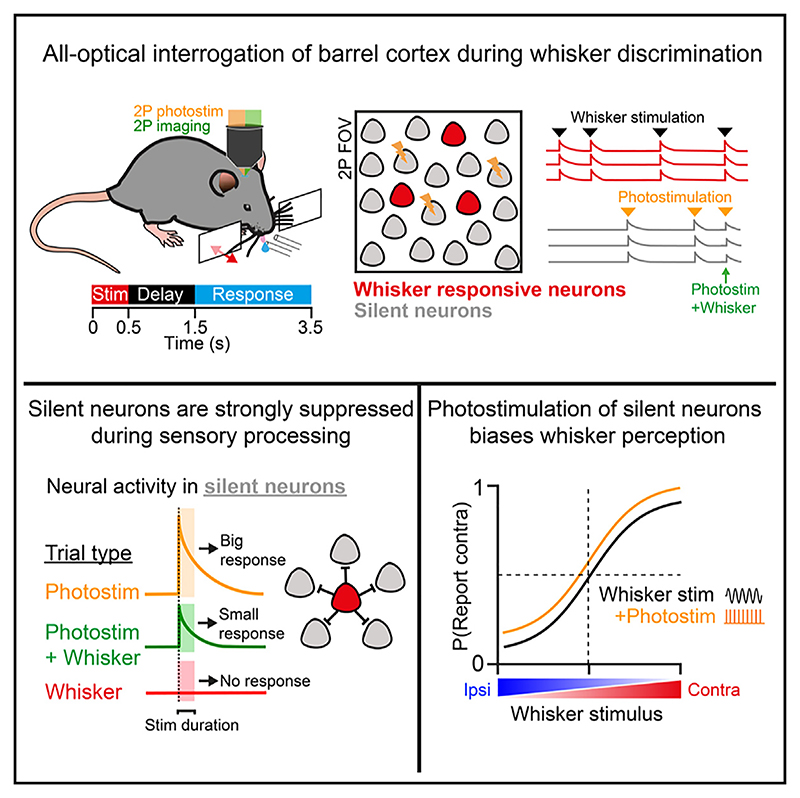

## Introduction

Understanding how sensory inputs are transformed into perceptual outputs requires functional dissection of cortical circuits during behavior.^1,2^ Neural circuits in the superficial layers of the sensory cortex are largely composed of excitatory neurons that show heterogenous stimulus tuning,^3^ structured patterns of connectivity,^4–8^ and low spike rates.^9^ GABAergic interneurons, which are densely connected with local excitatory neurons^10,11^ and have high baseline firing rates^12^ and broad receptive fields,^13^ provide inhibition that patterns spatiotemporal excitation.^14–18^ Accordingly, sensory processing in the superficial cortex is typically dominated by subsets of highly tuned neurons,^19–21^ with most excitatory neurons remaining “silent” during behavior.^22,23^

The observation that few neurons are engaged during sensory processing and that their activity correlates with perceptual decisions^24–27^ suggests the cortex uses a sparse neural code to generate stimulus percepts.^25,28,29^ Sparse coding is an efficient mechanism for encoding information,^30–32^ and has been observed experimentally across a range of neural systems.^33,34^ In causal support of the sparse coding hypothesis, experimental stimulation of small groups of cortical neurons, and even single neurons, can elicit perceptual responses.^26,35–41^ Moreover, functionally targeted microstimulation can also influence decisions in favor of the tuning of the manipulated neurons.^42–46^

As these findings indicate that perception is driven by small stimulus-tuned “ensembles,” the functional significance of the large proportion of non-responsive neurons in the sensory cortex has remained enigmatic.^23,47,48^ Neurons may appear silent if they have selective receptive fields that are not explored by standard experimental sensory stimulation paradigms.^33^ Alternatively, non-responsive neurons may be reserved for implementing circuit plasticity.^22,49^ Understanding why so few neurons respond strongly to sensory stimuli and how such sparse activity can drive reliable sensorimotor behavior is fundamental for understanding cortical circuit function.^21,50–52^

Here, we used simultaneous two-photon (2P) calcium imaging and holographic 2P photostimulation (PS)^53–56^ to interrogate sparse coding in the barrel cortex while head-fixed mice performed a bilateral whisker discrimination task. We opted to probe barrel cortex under bilateral sensory conditions as bilateral processing is an ethological aspect of tactile sensation for subterranean rodents.^57–60^ Moreover, perturbing barrel cortex impairs bilateral whisker tasks,^57,59,61^ suggesting that bilateral somatosensation is barrel cortex-dependent. By characterizing the circuit response to paired whisker stimulation and patterned 2P PS, we provide new insights into the mechanistic basis of sparse coding and show that intrahemispheric perceptual signals can be enhanced by releasing “non-coding” L2/3 neurons from inhibition, consistent with work implicating silent cortical neurons in sensorimotor plasticity.^22,49^

## Results

### A bilateral discrimination task for probing whisker perception

**Figure 1:**
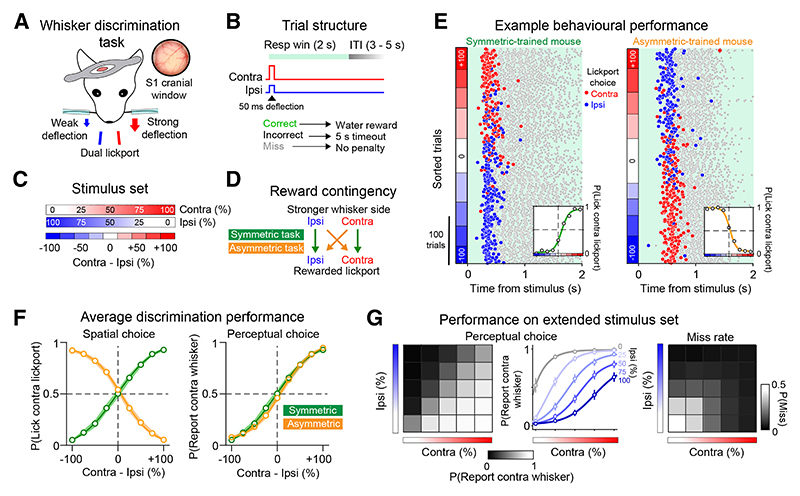
A whisker-guided task for graded bilateral intensity discrimination (A) Whisker discrimination task setup. The inset shows an image of cortex through a 3 mm cranial window implanted over S1. (B) Schematic of trial structure and trial outcome. (C) “Cross-fading” bilateral whisker deflection stimulus design. (D) Overview of symmetric (green) and asymmetric (orange) stimulus-reward contingencies. (E) Example session performance from a symmetric-trained mouse (left) and an asymmetric-trained mouse (right). Trials were delivered in a randomized order but were sorted along the y axis according to stimulus difference as in (C). Each row corresponds to a trial, and each marker corresponds to a lick. The first lick is colored red/blue for contra/ipsi choice. The inset shows the session psychometric curve. (F) Average psychometric performance for symmetric (green; *n* = 31 mice) and asymmetric (orange; *n* = 30 mice) trained mice. The left and right plots show spatial choice and perceptual choice tendency, respectively. (G) Average performance during matrix stimulus sessions. Left: average P(Report contra whisker) is shown across trial types with each square in the 5 × 5 grid representing a different combination of contra and ipsi input. Middle: behavioral data are replotted such that each row in the left behavioral matrix (corresponding to a different ipsi stimulus level) is now shown as a psychometric curve. Right: average miss rate is shown across stimuli. Group data in Figure 1 are shown as the mean across mice, with error bars representing SEM.

To probe the relationship between barrel cortex activity and stimulus perception, we developed a whisker discrimination task for head-fixed mice. First, we co-expressed the calcium indicator GCaMP6s and excitatory opsin C1V1 in barrel cortex neurons using a viral expression strategy (Figures S1A and S1B). During the task, the contralateral and ipsilateral C2 whiskers were simultaneously deflected, and mice discriminated the larger amplitude side and reported their choice with directional licking (Figures 1A–1C). We counterbalanced the stimulus-response contingency across mice (Figure 1D). Mice trained on the symmetric contingency learned a congruent spatial mapping between the stronger stimulus side and the target “lick” response (e.g., stim left → lick left), while mice trained on the asymmetric contingency learned the inverse rule (e.g., stim left → lick right). Mice learned the task structure through training on unilateral trials (Figures S2A and S2B). Performance was comparable across sides (Figure S2C) and contingencies (Figure S2D), although reaction times (RTs) were faster for symmetric-trained mice (Figure S2E). When using weaker stimuli, miss rate and RT increased (Figures S2F and S2G), and whisker-trimming abolished stimulus detection (Figure S2H). Unilateral muscimol infusion in barrel cortex selectively impaired contralateral trials (Figure S2I), indicating that performance requires both whisker input and barrel cortex.

Discrimination training yielded high-quality psychometric curves with large numbers of trials per session (309.1 ± 93.7 trials; mean ± SD; Figure 1E). Average psychometric curves from symmetric and asymmetric-trained cohorts of mice were inverted when we quantified spatial choice tendency as a function of stimulus difference (Figure 1F left), but comparable when we quantified the tendency mice would report the contralateral whisker stimulus (Figure 1F right). As the comparison between contingencies was not the primary focus of our study, we pooled data across contingencies unless otherwise stated. We trained some mice on an extended stimulus set comprising a larger combination of trial types (5 × 5 stimulus “matrix”; *n* = 83 sessions in 21 mice). Performance during these sessions demonstrates that mice solve the task by integrating stimuli bilaterally (Figure 1G). We also probed the temporal limits of bilateral discrimination (Figure S3A; *n* = 58 sessions in 25 mice). Temporal intervals led to strong choice biases that aligned with the leading stimulus side and saturated at 100 ms (Figures S3B and S3C). Temporal sensitivity appeared stronger in symmetric-trained mice (Figure S3D) and correlated with average RTs on whisker trials (Figure S3E). Together, our results demonstrate that the whisker system supports fine-scale discrimination of bilateral tactile input and that barrel cortex can generate stable sensory percepts within 100 ms of stimulus input to guide behavior.

### Bulk optogenetic activation of the barrel cortex evokes a contralateral percept

**Figure 2:**
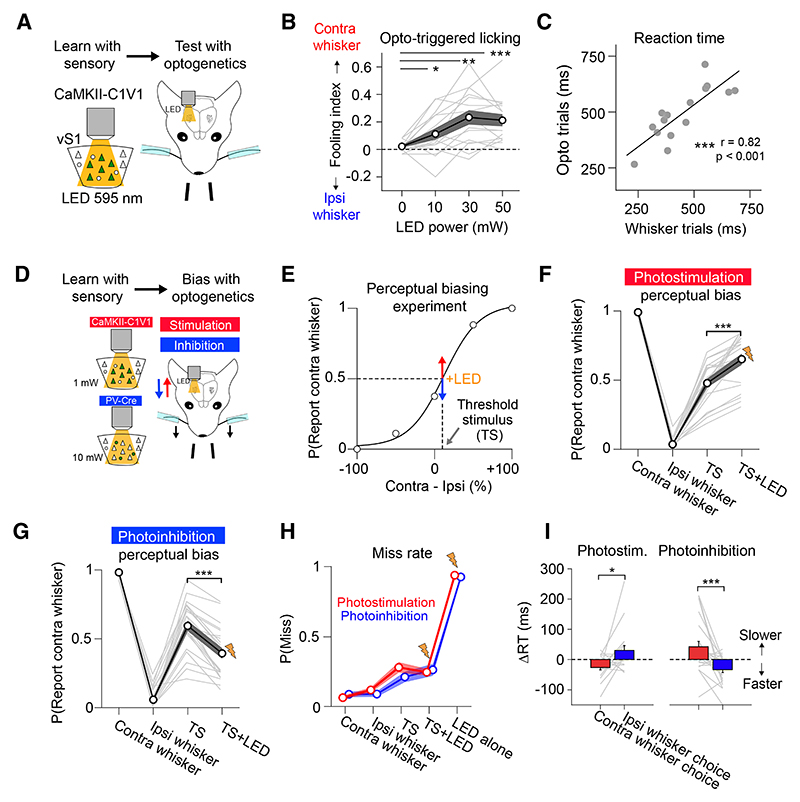
Optogenetic manipulation of barrel cortex during task performance (A) Optogenetic “substitution” experiment schematic. (B) Perceptual fooling index on optogenetic stimulation trials. (C) Correlation between average reaction times on optogenetic stimulation trials (mean across 30 and 50 mW trials) and whisker trials. Each data point shows an individual session. (D) Schematic of photostimulation (red) and photoinhibition (blue) perceptual biasing experiments. (E) Optogenetic stimuli were paired with the bilateral whisker threshold stimulus (TS). (F) Behavioral performance during photostimulation biasing experiments. (G) Behavioral performance during photoinhibition biasing experiments. (H) Miss rate is shown across trial types during photostimulation (red) and photoinhibition (blue) experiments. (I) Optogenetic biasing of TS trial reaction time for photostimulation (left) and photoinhibition (right) experiments. The mean difference in RT on trials where mice reported the contralateral whisker choice vs. the ipsilateral whisker choice is shown as red and blue bars, respectively. Data in Figure 2 show the average across sessions as circular markers and SEM (shaded error bars). Data from individual sessions are shown as thin gray lines. All statistical tests were two-tailed Wilcoxon signed-rank tests * *p* < 0.05; ** *p* < 0.01; *** *p* < 0.001.

Following learning, we photostimulated C1V1-expressing pyramidal neurons using an LED and measured whether a single optogenetic stimulus could “fool” mice into reporting a contralateral whisker deflection (Figure 2A). We calculated a “fooling index” as the difference in probability that optogenetic stimulation would evoke a contralateral vs. an ipsilateral whisker choice. Optogenetic stimulation of barrel cortex evoked illusory perceptual responses that increased with stimulation power (fooling index: 0 mW = 0.02 ± 0.04; 10 mW = 0.11 ± 0.16; * *p* = 0.047; 30 mW = 0.23 ± 0.2; ** *p* = 0.002; 50 mW = 0.21 ± 0.17; *** *p* = 6.1 × 10^−5^; *n* = 16 sessions in 12 mice; Figure 2B). Optogenetic stimulation evoked contralateral licking in symmetric-trained mice but ipsilateral licking in asymmetric-trained mice (Figure S4A). Mean RT on whisker and LED trials were highly correlated (Pearson’s correlation (r) = 0.82, *** *p* = 0.0002; Figure 2C), with faster LED-evoked RT measured in symmetric-trained mice (Figure S4B). LED-triggered licking was absent in control mice (Figure S4C). Our results therefore confirm that large-scale optogenetic activation of barrel cortex is sufficient to evoke contralateral whisker perception and initiate a goal-directed “action” specific to the learned sensorimotor context.^26,40^

### Bidirectional optogenetic biasing of whisker choice

Next, we assessed whether optogenetically manipulating barrel cortex biased perceptual choice at the whisker discrimination threshold (Figure 2D). We first performed PS experiments using a low LED power estimated to have negligible *de novo* perceptual saliency (1 mW; Figure S4D). At the start of each experiment, we calibrated the whisker threshold stimulus (TS; Figure 2E). LED stimulation biased psychophysical reports on TS trials toward the contralateral whisker (∆P[Report contra whisker] = 0.17 ± 0.13; *** *p* = 1.1 × 10^−4^; Wilcoxon signed-rank test; *n* = 21 sessions in 19 mice; Figure 2F), increasing contralateral lickport choices in symmetric-trained mice while increasing ipsilateral lickport choices in asymmetric-trained mice (Figure S4E). The effect was strongest when LED stimulation was delivered with a short delay relative to the TS (Figure S4F), which could reflect greater temporal coincidence between optogenetic and sensory-driven activity in cortex due to the short sensory signaling latency.

We then examined the effect of suppressing barrel cortex activity on choice tendency. We silenced barrel cortex by optogenetically activating (PV) interneurons expressing C1V1. Stimulation of PV interneurons simultaneously with TS input biased choice toward the ipsilateral whisker (ΔP[Report contra whisker] = −0.2 ± 0.11; *** *p* = 2.7 × 10^−5^; Wilcoxon signed-rank test; *n* = 23 sessions in 4 mice; Figure 2G). This effect decreased as a function of photoinhibition latency and was absent 100 ms after the TS (Figure S4G). During both PS and photo-inhibition experiments, LED presentation did not change miss rate on TS trials (PS TS vs. TS+LED ∆P[Miss] = −0.034 ± 0.13, n.s. *p* = 0.64; photoinhibition ∆P[Miss] = 0.05 ± 0.13, n.s. *p* = 0.09) and did not evoke reliable licking responses when presented alone (P[Miss] on LED trials; PS = 0.94 ± 0.06; photoinhibition = 0.93 ± 0.08; Figure 2H).

Optogenetic manipulations also influenced RT (Figure 2I). PS of excitatory neurons decreased RT for contralateral whisker choices (∆RT contra whisker choice = −26.4 ± 36.6 ms; ** *p* = 0.004; Wilcoxon signed-rank test), while also tending to increase RT for ipsilateral whisker choices (∆RT ipsi whisker choice = 30.3 ± 72.1 ms; n.s. *p* = 0.08; Figure 2I left). Photoinhibition of barrel cortex via activation of PV interneurons resulted in the inverse effect, increasing RT for contralateral whisker choices (∆RT contra whisker choice = 42.3 ± 85.2 ms; * *p* = 0.02) while decreasing RT for ipsilateral whisker choices (DRT ipsi whisker choice = −33.6 ± 45.7 ms; ** *p* = 0.004; Figure 2I right). RT effects also decreased as a function of LED onset latency (Figure S4H). Together, our experiments show that unilateral PS and photoinhibition exert inverse and timing-dependent biases in perceptual decisions and perceptual speed within 100 ms from whisker deflection onset, indicating that barrel cortex plays a causal, but transient, role in perceptual processing during task performance.

### 2P imaging of the barrel cortex during delayed discrimination

**Figure 3:**
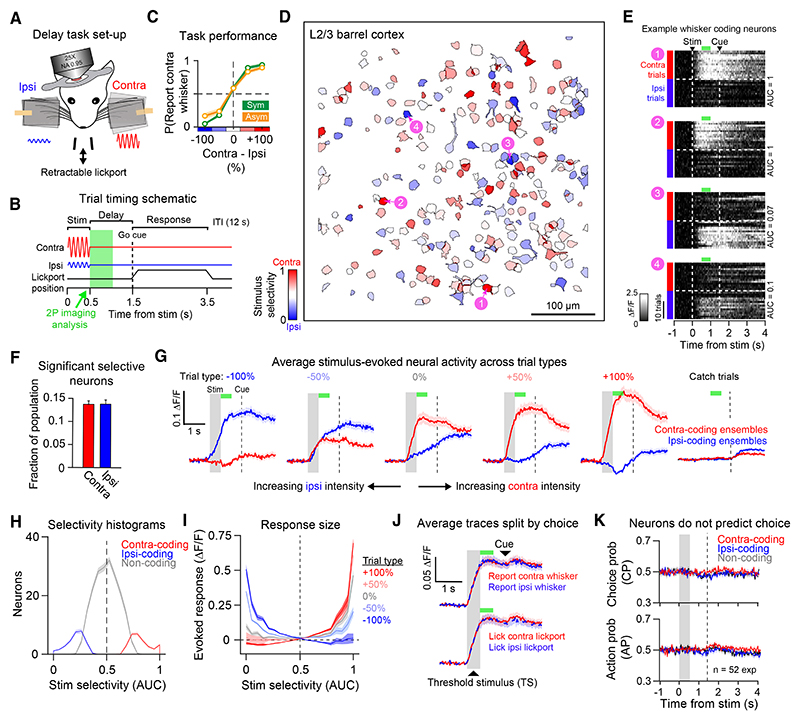
Characterization of task-evoked activity using two-photon calcium imaging (A) Schematic of the delayed-response discrimination task. (B) Delay-task trial structure. The green shading denotes the window used to analyze neural activity. (C) Average psychometric performance during imaging sessions for symmetric (green) and asymmetric (orange) trained mice. (D) Regions of interest (ROIs) corresponding to neuronal somata in an example FOV colored by stimulus selectivity. (E) Heat-maps showing sorted single-trial fluorescence responses on unilateral contra (red) and ipsi (blue) whisker trials for 4 example neurons numbered in (D). (F) Quantification of the mean fraction of significant contra-coding (red) and ipsi-coding (blue) neurons per FOV. (G) Average trial-evoked fluorescence traces for contra-coding neuron ensembles (red) and ipsi-coding neuronal ensembles (blue) across stimulus trial types in (C). (H) Histogram showing the average distribution of stimulus-selectivity scores for contra-coding (red), ipsi-coding (blue), and stimulus-non-coding (gray) neurons. (I) Evoked response amplitude across trial types shown as a function of stimulus selectivity. Colors correspond to different trial types as in (C) and (G). (J) Average fluorescence traces in contra-coding ensembles on TS trials split by perceptual choice (top) and spatial choice (bottom). (K) Average choice probability (CP; top) and action probability (AP; bottom) scores in contra (red), ipsi (blue), and non-coding (gray) ensembles across the trial. Data in Figure 3 are from 52 sessions across 13 mice (30 sessions in 7 symmetric-trained mice; 4.3 ± 2.0 (mean ± SD) sessions per mouse, and 22 sessions in 6 asymmetric-trained mice; 3.7 ± 1.8 (mean ± SD) sessions per mouse). Neural ensemble data were first averaged within session and then presented as the mean ± SEM across sessions.

To characterize task-related neural activity, we used 2P calcium imaging to record from GCaMP6s-expressing L2/3 neurons. As our initial experiments indicated that mice execute rapid decisions following whisker input, we developed a delayed-response version of the task to separate “sensation” and “action” epochs. To circumvent the requirement to trim the surrounding whiskers, we changed the stimulus effector from a glass capillary to a “paddle,” which engaged multiple whiskers simultaneously (Figure 3A) and should also drive a larger stimulus-responsive neuronal population. To further increase stimulus saliency to aid performance in this more demanding task, the whiskers were deflected using sinusoidal waveforms that were fixed in frequency (20 Hz) but varied in amplitude bilaterally across trials. Thus, mice solved the task by discriminating deflection amplitude bilaterally, analogous to the single-whisker task (Figure 1). Following stimulus presentation (500 ms), mice withheld licking across a 1 s delay until cued to respond with an auditory cue. A motorized lickport was then translated into position to initiate the response window (Figure 3B).

We trained two groups of naïve mice on the symmetric and asymmetric version of this delayed-response task (Figures S5A–S5F). Moving the stimulus paddles out of reach of the whiskers reduced performance to chance (Figure S5G), confirming that mice do not use audio-visual cues to solve the task. Following learning, mice were trained on the 5 × 5 matrix stimulus set (Figure S5H). Discrimination performance was comparable to the cohort of mice trained on the non-delay task (Figure 1G), indicating that the underlying perceptual discrimination task is similar between the single-whisker non-delay and multi-whisker delay tasks. We then imaged L2/3 during task performance (Figure 3C) while using simultaneous videography to quantify task-evoked movement and licking (Figure S6; STAR Methods). The 2P imaging field-of-view (FOV) was targeted to the stimulus paddle-responsive region of barrel cortex based on widefield fluorescence maps (Figures S1C and S1D). As we did not find clear differences in neural responses across symmetric and asymmetric-trained mice (Figure S7), we pooled both datasets unless otherwise stated.

### Sparse coding of whisker information in L2/3

To isolate sensory-evoked signals prior to licking, we analyzed stimulus-evoked fluorescence (∆F/F) responses during the delay epoch (Figure 3B *green shading*; 500–1,000 ms post-stimulus). We first assessed whether neurons showed a preference for contra vs. ipsi unilateral input by using receiver operating characteristic (ROC) analysis to compute stimulus selectivity (area under the curve; AUC). This revealed a broad distribution of selectivity across the L2/3 population, with some neurons preferring contra trials (red neurons in Figure 3D; neurons 1 and 2 in Figure 3E), and others preferring ipsi trials (blue neurons in Figure 3D; neurons 3 and 4 in Figure 3E). Surprisingly, a comparable proportion of neurons were significantly selective for contra (fraction of all cells: 0.14 ± 0.05 (SD); mean selectivity AUC 0.72 ± 0.04) and ipsi (fraction of all cells: 0.14 ± 0.07; mean selectivity AUC 0.31 ± 0.04) whisker stimulation (Figures 3F and S7A). We refer to neurons with significant selectivity as “stimulus-coding” and groups of stimulus-coding neurons in the FOV as ensembles.

Increases in stimulus information in stimulus-coding ensembles were time-locked to stimulus onset (Figures 3G and S8A), and stimulus selectivity was similar irrespective of trial outcome (Figure S8B). Outside of the stimulus presentation window, quantification of whisking did not predict contra vs. ipsi whisker stimulation (Figure S8D bottom), suggesting that active orofacial movements are similar across trial types. Unilateral response amplitude was larger in contra-coding ensembles (preferred-stimulus response (∆F/F): contra-coding ensembles = 0.18 ± 0.11; ipsi-coding ensembles = 0.12 ± 0.06; ** *p* = 0.003; Wilcoxon signed-rank test; 52 sessions), consistent with an intrahemispheric bias for contralateral input. Across bilateral trials, responses in stimulus-coding ensembles increased as a function of preferred-stimulus intensity (Figures 3G and S7C). Most neurons did not have a statistically significant stimulus preference (fraction of all cells: 0.72 ± 0.09; 183 ± 37.9 cells per FOV; mean AUC 0.5 ± 0.02; Figures 3H and S7B) and did not show reliable responses on average across the stimulus set (Figure 3I). We refer to these neurons as non-coding; however, they could code for variables not explored in the task. Our results therefore indicate that only a sparse subset of L2/3 neurons provide reliable stimulus-coding during task performance.

### L2/3 neurons do not predict categorical choice

During imaging sessions, we also included TS trials. As contra-coding ensembles showed stronger responses on bilateral trials, we assessed if contra-coding ensembles predicted choices on TS trials. Average activity patterns were similar on TS trials where mice reported different perceptual choices (Figure 3J top) and different spatial choices (Figure 3J bottom). To quantify this further, we calculated choice probability^24^ (CP). CP within stimulus-coding and non-coding ensembles remained at chance across the trial (Figure 3K top and S8E), with no clear correlation between stimulus selectivity and CP across the L2/3 population (Figure S8C top). We also did not find clear evidence of neuronal choice-coding in either symmetric or asymmetric-trained cohorts of mice (Figures S7A and S7D) and did not detect statistically significant choice neurons above the expected false positive rate (5%; Figure S8E). We then calculated a complementary metric we refer to as action probability (AP), with AP scores > 0.5 predicting contralateral lickport choices. Average AP scores also remained at chance (Figures 3K bottom and S8C bottom). We also did not detect significant spatial choice-predictive neurons above chance (Figure S8F).

However, a small proportion of neurons predicted if mice would report a decision vs. miss the trial (proportion of neurons: 0.07 ± 0.04 (mean ± SD); Figure S8G left). We summarized “lick” vs. “no lick” discrimination by calculating detect probability^62^ (DP). DP was significantly above chance in stimulus-coding ensembles (DP: contra-coding ensembles; 0.53 ± 0.11; ** *p* = 0.002; ipsi-coding ensembles 0.52 ± 0.1; ** *p* = 0.009) but not in non-coding ensembles (DP in non-coding ensembles; 0.5 ± 0.09; n.s. *p* = 0.32). However, performing the same ROC analysis on videography-extracted movement traces revealed that lick trials could also be predicted from increases in whisking (Figure S8G bottom). Thus, it is possible that neural DP signals are caused by non-instructed preparatory movements prior to licking. In comparison, whisking did not predict contralateral perceptual choice (Figure S8E bottom) or contralateral lickport choice (Figure S8F bottom). However, lateralized whisking did align with spatial choice during the choice window, consistent with an increase in ipsilateral orofacial movement during directional licking (Figure S8F bottom). Thus, although barrel cortex showed sparse coding of stimulus information, we did not find a consistent predictive relationship between neural responses, or task-evoked movements, and categorical choice.

### Targeted 2P PS of L2/3 neurons during behavior

**Figure 4:**
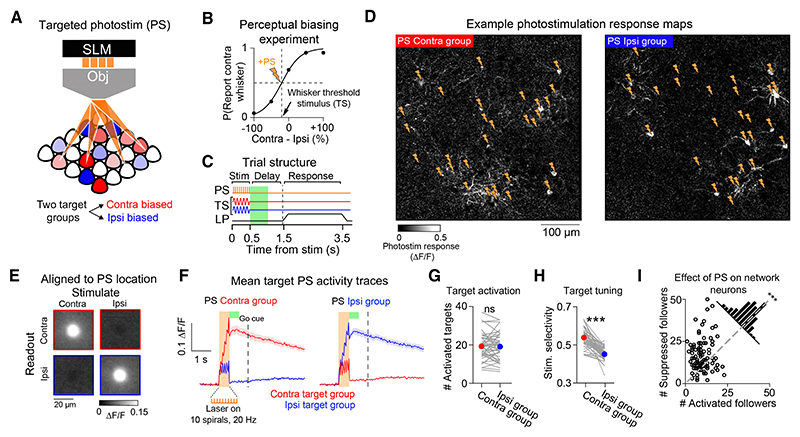
Two-photon photostimulation of L2/3 neurons during whisker discrimination (A) SLM-targeted two-photon photostimulation (PS) of contra vs. ipsi-biased L2/3 ensembles. (B) Photostimulation was delivered simultaneously with the whisker threshold stimulus (TS). (C) Trial structure for paired sensory and photostimulation (TS + PS) trials. The green-shaded region shows the 2P imaging analysis window. (D) Photostimulation response maps showing the mean response 500–1,000 ms post-stimulus from an example session. Stimulation of the contra and ipsi target groups is shown on the left and right, respectively, with lightning bolts indicating the targeted locations. (E) Average pixel-wise PS response maps centered on all PS spiral sites for contra (top row) and ipsi (bottom row) SLM targets, on contra group stimulation trials (left column) and ipsi group stimulation trials (right column; average across 1,560 target sites across 52 session). (F) Average fluorescence traces from contra (red) and ipsi (blue) target neurons on photostimulation trials. The orange bar indicates the photostimulation duration, and the green bar shows the response analysis window. Data are averaged across 52 sets of target groups from 52 sessions across 13 mice; 1,992 activated target neurons in total. (G) Quantification of the number of target neurons activated by photostimulation during TS + PS trials. The colored marker shows the mean, and gray lines show individual session data. (H) Same as in (G), but showing quantification of the average stimulus selectivity across activated target groups. (I) Quantification of the number of suppressed vs. activated network followers during TS + PS trials. 52 sets of followers in 52 sessions; 13 mice; 1,299 activated and 2,078 suppressed follower neurons in total. Statistical comparisons were made across sessions with Wilcoxon signed-rank tests. * *p* < 0.05, ** *p* < 0.01; *** *p* < 0.001.

Our experiments in the non-delay task show that manipulating barrel cortex drives lateralized biases in psychophysical performance (Figure 2). However, the number, functional identity, and layer-specificity of neurons “activated” by one-photon optogenetic stimulation are poorly controlled, making it unclear which neurons are involved in driving the behavioral effect. We therefore designed a more powerful all-optical experiment^37,53,54^ using 2P targeted PS to test how targeted stimulation of different L2/3 ensembles impacts perceptual choice (Figures 4A–4C). Following task imaging (Figure 3), we selected two groups of 30 pyramidal neurons in the FOV co-expressing GCaMP6s and soma-targeted C1V1 (st-C1V1, Figure S1D) for holographic PS (Figure 4D). We designed target groups with the intention that one would have a contra whisker-selectivity bias, while the other an ipsi bias, by selecting the 30 PS-responsive neurons with the highest and lowest stimulus-selectivity scores, respectively (Figures S9A–S9C). However, as neurons with strong responses to both PS and sensory input were rare (Figures S9D and S9E), this approach likely includes a large number of neurons with weak selectivity. PS was performed using a spatial light modulator (SLM^63–66^; Figure 4A) and a galvanometer-based spiral scanning strategy^53^ (STAR Methods). PS-evoked responses were quantified using simultaneous 2P calcium imaging. We paired PS with the whisker TS (TS + PS; Figure 4C) and randomized PS across contra and ipsi-biased target groups across trials.

We parsed neurons into “target” and “network” categories based on lateral somatic proximity to the nearest PS spiral site (Figures S10A–S10D). PS evoked soma-shaped increases in fluorescence at the intended spatial locations in the FOV (Figures 4D and 4E) and stimulation-locked fluorescence increases in the corresponding target groups (Figure 4F). We refer to neurons in target “zones” with significant activity increases on PS trials as activated targets. Although we targeted 30 neurons in each experiment, not all of them responded with significant increases in activity (# activated targets in TS + PS contra group 19.3 ± 6.5; mean ± SD; # activated targets in TS + PS ipsi group = 19.1 ± 6.8; n.s. *p* = 0.72; Wilcoxon signed-rank test; *n* = 52 sessions; Figure 4G). The number of activated targets on PS and TS + PS trials was highly correlated (Figure S10E) and was also correlated with the number of PS-responsive neurons in the FOV, as expected (Figure S10F). The number of activated targets was also closely related to the total number of neurons located within target zones’ (Figure S10G), which we did not control for experimentally. The activated target groups had a weak, but consistent, whisker stimulus-selectivity bias (mean selectivity difference across groups = 0.083 ± 0.063, *p* = 5.× 10^−9^; Figure 4H). We also quantified the impact of targeted PS on non-targeted “follower” network neurons. Across experiments, we found that a larger number of followers were suppressed on TS + PS trials (# activated followers: 12.5 ± 5.6; # suppressed followers: 20 ± 11.5; *** *p* = 8.9 × 10^−8^; Wilcoxon signed-rank test; *n* = 104 PS conditions; 52 sessions; Figure 4I). Thus, patterned 2P PS resulted in the activation of neurons within target zones while predominantly suppressing neurons in the local L2/3 network.

### Perceptual bias scales with the number of photostimulated L2/3 neurons

**Figure 5:**
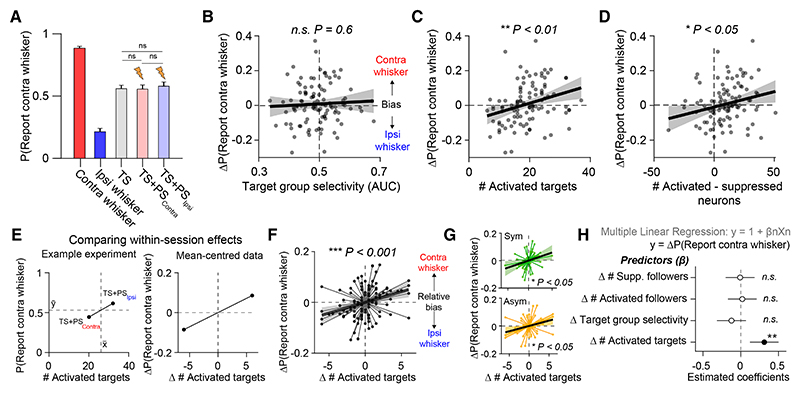
The number of activated target neurons predicts perceptual bias (A) Quantification of average perceptual choice tendency during the targeted photostimulation experiment. (B) Comparison of perceptual bias and target group whisker selectivity. (C) Comparison of perceptual bias and the number of activated target neurons. (D) Comparison of perceptual bias and the net change in population activity. (E) Within-session differences across photostimulation conditions were compared by “mean-centering” the data points from each session. This procedure is shown with a single example session. The dashed lines on the left plot indicate the mean P(Report contra whisker) (horizontal) and target response (vertical) across the two conditions. (F) Correlation between within-session difference in the number of activated targets and perceptual bias across all mean-centered data points. Data points corresponding to the same session are joined with a thin line that passes through the origin. (G) The same as in (F) but split by sessions from symmetric (green; top) and asymmetric (orange; bottom) trained mice. (H) A multiple linear regression (MLR) model summarizing the relationship between target and network photostimulation predictors and within-session perceptual bias. Statistically significant predictors are shown with black markers, with error bars indicating 95% coefficient confidence intervals. Data in Figure 5 are from 52 sessions across 13 mice. Each data point represents an individual photostimulation condition (one for TS + PS contra trials and one for TS + PS ipsi trials), with a total of 104 data points. Total number of control TS trials = 2,374, total number of TS trials with PS = 2,403. In correlation plots, shaded error bars denote the 95% confidence bounds of a linear regression fit to the data (black line).

PS of small ensembles did not result in average changes in choice tendency on TS trials (P[Report contra whisker] on TS trials: 0.56 ± 0.2; 45.7 ± 21.7 trials; mean ± SD; on TS + PS contra trials: 0.56 ± 0.24; 22.8 ± 10.5 trials; n.s. *p* = 0.68; on TS + PS ipsi trials: 0.58 ± 0.24; 23.4 ± 10.8 trials; n.s. *p* = 0.33; Wilcoxon signed-rank test; *n* = 52 sessions; Figure 5A). Across sessions, target group whisker selectivity also did not predict perceptual bias (Pearson’s corr (r) = 0.05; n.s. *p* = 0.06; Figure 5B). Instead, we found that perceptual bias significantly correlated with the number of activated target neurons (Pearson’s corr (r) = 0.3; ** *p* = 0.002; Figure 5C). This correlation was maintained when using different statistical thresholds for defining activated targets (Figure S11A) and was absent when we repeated our analysis on resampled control TS trials (Figure S11B). Moreover, target groups did not show CP on control trials that differed from chance (Figure S11C), and target group CP did not predict the impact of PS on behavior or the number of activated targets across sessions (Figure S11D).

Intriguingly, activating low numbers of targets appeared to bias choice toward the ipsilateral whisker (Figure 5C). By quantifying the number of activated vs. suppressed neurons across both targets and followers, we found that perceptual bias also correlated with net change in population activity (Pearson’s corr (r) = 0.23; * *p* = 0.02; Figure 5D). This suggests that ipsilateral biases tended to occur in sessions where PS was comparatively weak, and thus the net effect on the L2/3 circuit was slightly suppressive (Figure 5D). However, quantification of follower neurons alone was not sufficient to predict changes in behavior (Figure S11E), suggesting that the impact of PS on perceptual bias cannot be wholly explained by differences in network state.

### Within-session perceptual bias correlates with the number of activated target neurons

As we stimulated two target groups per session, we then analyzed whether differences in the number of activated target neurons explained variability in within-session perceptual bias. To examine this effect, we mean-centered the data points from the two PS conditions from each session (Figure 5E). Across the mean-centered dataset, variability in the number of activated targets also significantly correlated with relative perceptual bias (Pearson’s corr (r) = 0.33; *** *p* = 0.0007; Figure 5F). This correlation was present in both symmetric (Pearson’s corr (r) = 0.33; * *p* = 0.011; *n* = 30 sessions, 7 mice; Figure 5G Top) and asymmetric-trained cohorts of mice (Pearson’s corr (r) = 0.34; * *p* = 0.025; *n* = 22 sessions, 6 mice; Figure 5G bottom). We used a multiple linear regression model to summarize within-session effects of PS on perceptual choice bias (Figure 5H). Perceptual bias was significantly predicted by the number of activated targets (estimated coefficient = 0.31; t-stat = 3.3; ** *p* = 0.001) but not the number of activated (estimated coefficient = 0.03; t-stat = 0.32; n.s. *p* = 0.74) or suppressed (estimated coefficient = −0.01; t-stat = −0.5; n.s. *p* = 0.96) follower neurons. Perceptual bias was also not correlated with target group whisker stimulus selectivity (estimated coefficient = −0.16; t-stat = −1.6; n.s. *p* = 0.1). Additional analysis confirmed that neither perceptual bias nor the number of activated target neurons correlated with differences in pre-stimulus network fluorescence or peri-stimulus whisking or body movement across PS trial types (Figure S11F). Thus, our results indicate that sparse unilateral manipulation of the L2/3 circuit during bilateral discrimination causally drives a lateralized perceptual bias that scales with the number of neurons activated.

### Activation of task-silent target neurons predicts perceptual bias

**Figure 6:**
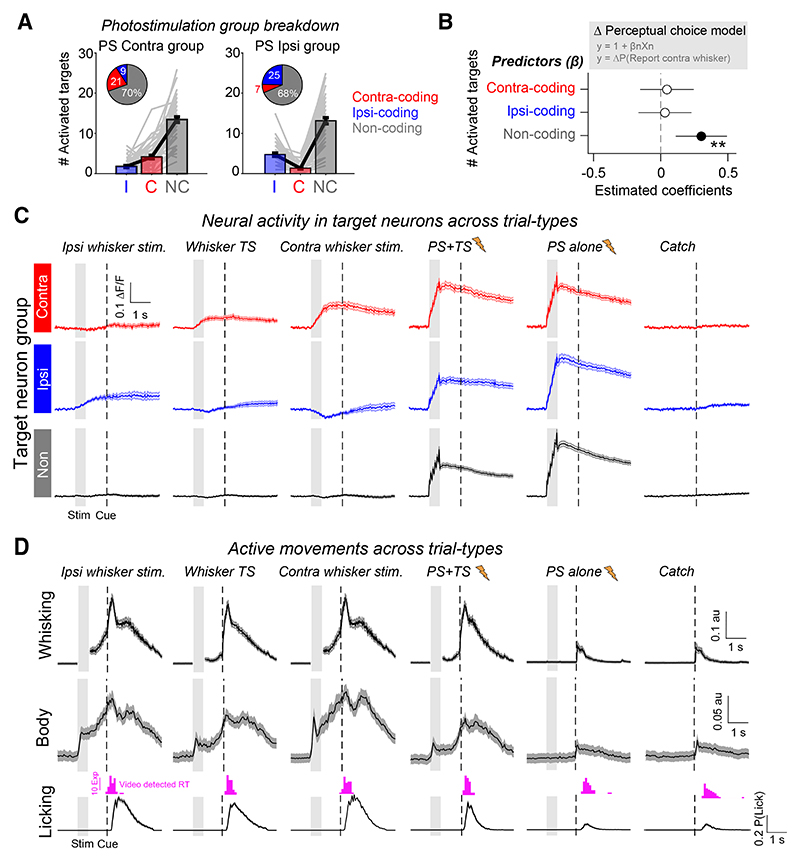
Photostimulation of non-coding neurons predicts perceptual bias (A) Quantification of the number of contra-coding (red), ipsi-coding (blue), and whisker non-coding (gray) activated target neurons that made up the two photostimulation target groups. Individual lines show individual sessions. The inset circle charts indicate the average proportional group summary. (B) A multiple linear regression (MLR) model summarized the relationship between the number of photostimulated stimulus-coding and non-coding neurons and perceptual bias. Marker points shown the estimated coefficients with error bars indicating the 95% confidence intervals. (C) Trial-evoked activity traces from contra-coding (red), ipsi-coding (blue), and non-coding (gray) photostimulation target neurons are shown across different trial types. (D) The time course of average trial-evoked contralateral whisking (top), body movement (middle), and licking (bottom) behavioral measures are shown across trial types. The magenta histogram shows the distribution of mean reaction times assessed using videography. Note that during whisker stimulation (gray shading), we do not plot whisking traces as it is unclear which whisker movements are driven by the stimulus and which are the result of active movement. Data in Figure 6 are from 52 sessions in 13 mice. Total number of neurons analyzed: 274 contra-coding targets; 329 ipsi-coding targets; and 1,381 non-coding targets. Data are shown as the mean and SEM across sessions.

Our findings indicate that the perceptual effect of L2/3 PS during our task is predicted by the number, and not tuning, of activated target neurons (Figures 5B and 5H). However, although the target groups did show an average stimulus-selectivity bias (Figure 4H), additional analysis revealed that the majority of activated targets did not have a statistically significant whisker stimulus preference (Figure 6A). As stimulation predominantly activated non-coding target neurons in the circuit, we assessed whether this was sufficient to predict the perceptual effect of targeted PS. A multiple linear regression analysis (Figure 6B) confirmed that the number of activated non-coding target neurons significantly predicted perceptual bias (estimated coefficient = 0.3; t-stat = 3.14; ** *p* = 0.002). In comparison, neither the number of activated contra-coding targets (estimated coefficient = 0.05; t-stat = 0.46; n.s. *p* = 0.64) nor the number of ipsi-coding targets (estimated coefficient = 0.03; t-stat = 0.32; n.s. *p* = 0.75) predicted the behavioral effect. The correlation between non-coding target neuron activation and perceptual bias was present across sessions (Pearson’s corr (r) = 0.31; ** *p* = 0.001; Figure S12A) and within a session (Pearson’s corr (r) = 0.21; * *p* = 0.03; Figure S12B).

Non-coding target neurons showed reliable PS-evoked responses but did not show whisker-evoked activity (Figure 6C). This was consistent for non-coding target neurons in the contra-biased and the ipsi-biased target groups (Figure S13). Analysis of behavioral videography also revealed that trials with whisker stimulation evoked sustained increases in active whisking, body movement, and licking (Figures 6D and S6C). As fluorescence in non-coding target neurons remained at pre-stimulus levels throughout the trial epoch, this indicates that the non-coding target neurons are also not significantly activated by task-evoked movements. Our findings therefore indicate that small numbers of “task-silent” L2/3 neurons, which show no clear functional relationship to sensory, motor, or reward variables during task performance, can influence perceptual decisions if recruited into the active population through targeted optogenetic manipulation.

### Patterned stimulation reveals the specificity of L2/3 inhibition during whisker processing

**Figure 7:**
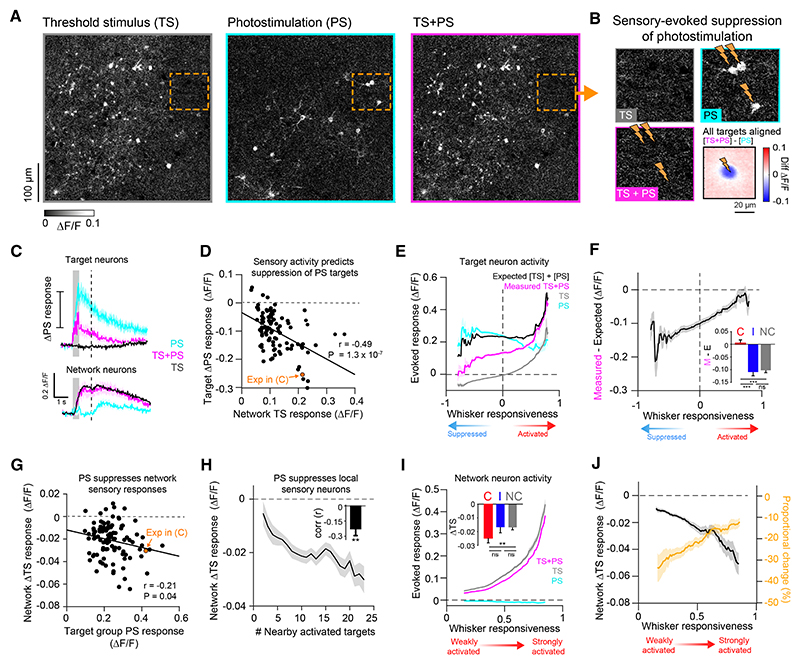
Patterned photostimulation reveals potent inhibitory pressure in L2/3 during task performance (A) Response maps for threshold stimulus (TS; gray), photostimulation (PS; cyan), and combined TS and PS (magenta) trials for an example session. (B) Close up of the orange-bounded region shown in (A). Lightning bolts indicate the location of PS targets. Bottom right shows the average pixel-wise fluorescence difference across TS + PS and PS trials center-aligned on all photostimulation target locations. (C) Extracted fluorescence traces from target neurons (top; *n* = 23 neurons) and TS-responsive network neurons (bottom; *n* = 57 neurons) across trial types from the example session in (A). (D) Suppression of photostimulation responses in the target neuron group on PS vs. TS + PS trials is correlated with the mean response to TS whisker trials in non-targeted network cell group. Each data point represents a single photostimulation condition. (E) Responses in photostimulation target neurons across different trial types are plotted as a function of whisker responsiveness. (F) The difference between the measured (magenta in E) and expected (black in E) response to combined TS and PS stimuli in target neurons is plotted as function of whisker responsiveness. The inset shows quantification of this difference averaged across contra-coding (red), ipsi-coding (blue), and non-coding (gray) target neuron ensembles. (G) Suppression of TS responses in network neuron groups on TS + PS trials is plotted against average PS-evoked responses in target neuron groups. (H) Average PS-evoked suppression of TS responses in network neurons is binned as a function of the number of nearby activated target neurons (counted within a 200 μm radius). The inset shows the average Pearson’s correlation coefficient between sensory suppression and number of nearby targets across sessions (r = −0.21 ± 0.47; mean ± SD; ** *p* = 0.002 Wilcoxon signed-rank test tested against 0). (I) Responses in TS-responsive network neurons across trial types are plotted as a function of whisker responsiveness. The inset shows quantification of the change across TS and TS + PS trials with respect to ensemble groups as in (F). (J) The difference in network neuron response on TS and TS + PS is shown as a function of whisker responsiveness. The absolute change is shown in black, and the proportional change relative to TS baseline is shown in orange. Data in Figure 7 come from 104 photostimulation conditions across 52 sessions in 13 mice. Total number of target neurons analyzed = 1,992; total number of network neurons analyzed = 2,429. Total number of trials analyzed: 3,466 TS, 3,568 PS; 3,447 TS + PS trials. Data are shown as the mean and SEM across sessions. Statistical comparisons were Wilcoxon signed-rank test. n.s. *p* > 0.05; * *p* < 0.05; ** *p* < 0.01; *** *p* < 0.001.

Comparing target responses across trial types revealed that PS responses were notably larger in the absence of concurrent whisker stimulation, particularly in ipsi-coding and non-coding target neurons (Figure 6C). This prompted us to examine the interaction between sensory and PS-triggered activity patterns (Figure 7A). For some targets, PS responses were near abolished on TS + PS trials, and all PS target locations showed a reduction in fluorescence when comparing PS trials with TS + PS trials (Figure 7B). To assess the relationship between the decrease in PS response and the TS-evoked sensory response, we quantified the average difference in the target group response on PS and TS + PS trials and compared this with the average response to TS input in the TS-responsive non-targeted network neurons (Figure 7C). Across sessions, the change in target group PS response was negatively correlated with the network response to sensory input (Pearson’s corr (r) = −0.49; *** *p* = 1.× 10^−7^; Figure 7D). This indicates that the stronger the whisker-evoked response in the network, the stronger the suppressive effect on PS-evoked activity in the target neurons.

To examine whether this effect showed specificity at the single neuron level, we quantified fluorescence responses across trial types in all activated targets as a function of whisker responsiveness (Figure 7E). We defined whisker responsiveness as the AUC score from an ROC analysis comparing TS with catch trial responses and adjusted scores between −1 and 1 such that the sign denoted negative vs. positive modulation by TS input. We then calculated the difference between measured TS + PS responses and the expected linear sum of separate TS and PS trial-evoked responses as an approximation for sensory-induced suppression of PS activity (Figure 7F). Target neurons with high whisker responsiveness scores appeared largely unaffected by sensory-induced network suppression. By contrast, neurons with lower whisker responsiveness scores and those with negative scores showed a large difference. When quantifying this effect with respect to stimulus-coding groups, we found that contra-coding targets groups showed no difference between measured and expected response (0.004 ± 0.01; n.s. *p* = 0.78), whereas both ipsi-coding target groups (−0.11 ± 0.11; *** *p* = 2.6 × 10^−8^) and non-coding target groups (−0.1 ± 0.07; *** *p* = 6.× 10^−10^) showed a strong suppression of PS activity on TS trials (Figure 7F *inset*). Thus, patterned PS revealed recruitment of potent cortical inhibition in L2/3 during whisker processing that appeared to preferentially impact ipsi-coding and non-coding neurons in the L2/3 circuit.

### Patterned stimulation suppresses local whisker-evoked signals

We next investigated the impact of patterned PS on whisker-evoked responses in the local circuit by analyzing TS-responsive neurons that were not targeted for PS. On average, targeted PS reduced the amplitude of the TS-response in network neurons (∆TS response = −0.02 ± 0.015 ∆F/F; *** *p* = 9.7 × 10^−18^, −19.4 ± 13.6% proportional change; *n* = 104 PS conditions, 52 sessions). This reduction was correlated with the amplitude of PS response in the target groups (Pearson’s corr (r) = −0.21; * *p* = 0.04; Figure 7G). The magnitude of sensory response suppression was stronger for network neurons in close proximity to large numbers of PS-activated target neurons (Figure 7H), suggesting that the suppressive effect of PS on the local circuit decreases with distance. All non-targeted TS-responsive neurons showed reduced TS responses on TS + PS trials on average (Figure 7I), with those that were part of contra-coding ensembles tending to show larger absolute changes in ∆F/F (Figure 7I *inset*). However, neurons with larger whisker responsiveness scores showed smaller proportional changes relative to the baseline amplitude of the TS-evoked response (Figure 7J). As strongly whisker-responsive neurons were relatively less inhibited by network-evoked suppression, this indicates that global inhibition may serve an important role in selectively eliminating weak responses in the circuit.

### Competitive interactions between contralateral and ipsilateral whisker signals in L2/3

**Figure 8:**
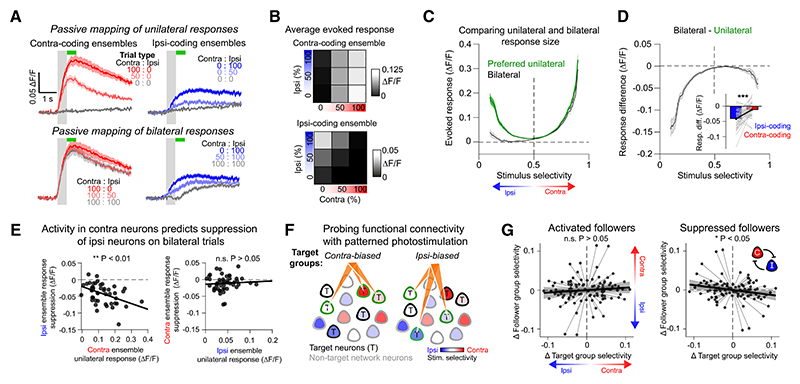
Antagonistic interactions between contra and ipsi-coding neurons in L2/3 (A) Fluorescence traces in contra-coding (left; red) and ipsi-coding (right; blue) ensembles across unilateral (top) and bilateral (bottom) whisker deflection intensities (%). (B) Quantification of the mean fluorescence responses across a × 3 matrix stimulus set in contra-coding (top) and ipsi-coding (bottom) ensembles (average ensemble response across 52 sessions). (C) Comparison of responses to preferred unilateral whisker stimulation (green; 100% unilateral stimulation) and matched bilateral whisker stimulation (black; 100% bilateral stimulation) plotted as a function of stimulus selectivity. (D) The difference in unilateral and bilateral responses (shown in C) is plotted as a function of stimulus selectivity. The inset shows quantification of this difference in contra and ipsi-coding ensembles (*n* = 52 sessions; Wilcoxon signed-rank test; *** *p* < 0.001; thin gray lines show individual sessions). (E) Comparison of response suppression in one ensemble group on bilateral trials with the unilateral response in the other ensemble group. Each marker point represents an individual session, 52 sessions in total. Total number of contra-coding neurons analyzed 1,907; ipsi-coding neurons 1,925. (F) Probing functional connectivity in the circuit using targeted photostimulation of whisker-biased target groups. (G) Comparing average whisker selectivity of network followers in response to targeted photostimulation. Correlations are measured across 104 mean-centered data points (2 photostimulation conditions per session, 52 sessions in total). Shaded error bars denote the 95% confidence bounds of a linear regression fit to the data.

Finally, we performed additional experiments to examine inter-actions between contra and ipsi-evoked signals in L2/3. After the targeted PS experiment, we mapped passive neuronal responses to a “× 3” stimulus set of unilateral and bilateral stimuli (Figures 8A and 8B). Contra-coding ensembles, defined based on activity during the behavioral experiment, showed large responses to contralateral stimulation that appeared robust to concurrent ipsilateral whisker stimulation (Figure 8B top). By contrast, ipsilateral-evoked signals in ipsi-coding ensembles were weaker and appeared strongly suppressed by concurrent contralateral stimuli (Figure 8B bottom). We compared responses to the preferred unilateral stimulus (100% intensity) to bilateral stimulation (100% intensity on both sides) as a function of stimulus selectivity (Figure 8C) and calculated the difference between the bilateral and preferred unilateral response (Figure 8D). Contra-coding ensembles showed a modest reduction in response on bilateral stimulation trials (change in response (∆F/F): −0.01 ± 0.02; ** *p* = 0.001; Wilcoxon signed-rank test; *n* = 52 sessions), whereas ipsi-coding ensembles showed stronger attenuation (change in response (∆F/F): −0.04 ± 0.03; *** *p* = 3.7 × 10^−9^; Figure 8D *inset*). The difference between contra and ipsi-coding ensemble suppression was significant (*** *p* = 1.9 × 10^−5^).

Across sessions, bilateral suppression of the ipsilateral ensemble was correlated with the contra-coding ensemble response to unilateral contralateral stimulation (Pearson’s corr (r) −0.44; ** *p* = 0.002; Figure 8E left). The inverse relationship between contra-coding ensemble suppression and the ipsi-coding ensemble response was not significant (Pearson’s corr (r) 0.07; n.s. *p* = 0.66; Figure 8E right). This indicates that contra-evoked signals may dominate during bilateral whisker processing by suppressing ipsi-responses in the circuit. To directly examine this relationship, we performed additional analysis of our PS dataset to assess whether the whisker selectivity of the PS target groups correlated with the selectivity of recruited follower neurons. For each session, we restricted our analysis to neurons that were not part of either activated target group, and thus, we could directly compare the local circuit response to two different photostimuli (Figure 8F). Across the dataset, variability in target group whisker selectivity did not correlate with variability in the average whisker selectivity of the activated followers (Pearson’s corr (r) = 0.1; n.s. *p* = 0.32; Figure 8G left). However, target group whisker selectivity negatively correlated with the selectivity of the suppressed followers (Pearson’s corr (r) = −0.23; * *p* = 0.018; Figure 8G right), indicating that stimulation of L2/3 contra-coding neurons tends to suppress ipsi-coding neurons in the FOV and vice versa. Thus, our findings demonstrate that strong competitive interactions between different L2/3 ensembles dominate sparse cortical dynamics and provide evidence that mutual inhibition between contra and ipsi-coding ensembles may be a prominent feature of intrahemispheric L2/3 activity during cross-hemispheric sensory processing.

## Discussion

We combined 2P calcium imaging and 2P PS to probe sparse coding in the barrel cortex during a challenging discrimination task. We show that sparse coding of whisker stimuli is ensured by preferential sensory-evoked inhibitory suppression of non-coding neurons, which helps to ensure a high signal-to-noise ratio for the detection of small perturbations. Moreover, although contralateral and ipsilateral whisker signals were represented in sparse subsets of stimulus-coding L2/3 neurons during task performance, the effect of intrahemispheric PS on lateralized perceptual bias depended on the number of non-coding target neurons activated. This suggests that non-coding neurons can be engaged to enhance sensory processing, which could be achieved endogenously via neuromodulation^67,68^ or cortical plasticity.^22,49^

### A whisker discrimination task to probe tactile perception

We developed a behavioral task framework suitable for probing the link between neural circuit activity in sensory cortex and behavior.^69^ Through both gain- and loss-of-function perturbations, we demonstrate that the perceived intensity of contralateral whisker input is causally linked to barrel cortex activity. By manipulating the bilateral stimulus interval in the non-delay task, we also found that unilateral percepts were formed rapidly and showed robustness to late-arriving distraction characteristic of choice-related attractor state dynamics.^70^ The barrel cortex-dependent sensory integration window, which we estimate to be ~100 ms based on temporal discrimination and photoinhibition experiments, is comparable to cortical processing windows in other whisker tasks^26,71,72^ and tasks using other sensory modalities.^73^ It is also consistent with findings showing that whisker input and microstimulation-induced activity quickly spread from barrel cortex to high-order sensory and motor areas,^74,75^ and demonstrates that barrel cortex plays a causal role in initiating rapid sensorimotor transformations during goal-directed behavior. Despite coding stimulus information, we did not find choice signals in barrel cortex. However, choice could be represented in membrane potential dynamics^26^ or temporal spike patterns,^76^ which may be difficult to resolve using calcium imaging, or in deeper cortical layers, which we did not sample during our experiments. Alternatively, decision-related processing may occur downstream from S1 in areas that also integrate bilateral whisker input, such as striatum^77^ and/or S2.^78,79^ Future work is required to investigate the downstream circuits implicated in the decision process to further elucidate the role of barrel cortex in coordinating cross-hemispheric perceptual behavior.

### Interhemispheric processing in the barrel cortex

Cross-hemispheric projections are a prominent feature of many cortical circuits.^80–83^ Surprisingly, we find that L2/3 contains equal proportions of ipsilateral and contralateral whisker-selective neurons, and also that strong competitive interactions between bilateral tactile signals occur at the level of primary somatosensory cortex during perceptual decision-making. This extends previous work characterizing bilateral responses in barrel cortex, which has predominantly been performed under anesthesia or in non-behaving states.^79,84,85^ Although ipsilaterally tuned neurons showed robust activity on unilateral trials, they were strongly attenuated by concurrent contralateral whisker stimulation. These findings support previous evidence that the short cross-callosal latency makes ipsilateral signals susceptible to rapid feedforward recruitment of cortical inhibition by contralateral-evoked thalamocortical input during bilateral stimulation,^84–88^ further indicating that the integration of bilateral tactile signals may relate to the precise spatiotemporal sequence of whisker stimulation.^84,85^

Our behavioral experiments also indicate that competition between hemispheres plays an important role in task-related perceptual processing. For example, results from the one-photon optogenetic-biasing experiments (Figure 2I) show that manipulating one hemisphere has an equal but opposite effect on the reaction time for the non-stimulated hemisphere. The push-pull direction of this bias switches depending on whether one delivers unilateral photoinhibition or photoactivation, providing strong causal evidence that both hemispheres have the capacity to directly compete during whisker-guided behavior.^89–92^ In rodent cortex, several mechanisms can mediate inhibitory interactions between hemispheres. These include transcallosal feedforward recruitment of local PV inter-neurons,^79,83,92,93^ recruitment of interneurons in superficial layers, which subsequently inhibit the distal dendrites of neurons in deeper layers,^91^ as well as via long-range callosally projecting inhibitory neurons.^93^ The extent to which these different inhibitory pathways are engaged in parallel or differentially under stimulus-specific or task-specific conditions remains unclear. Moreover, callosal interactions also mediate more refined functions, including the homotopic transfer of sensorimotor signals and learned information,^82,94,95^ and shaping receptive fields and circuit plasticity.^89,90,96^ Future work is therefore needed to elucidate how organization of callosal microcircuits shapes bilateral somatosensation.

### Intracortical inhibition enforces sparse coding in L2/3

Our experiments revealed strong antagonism between contralateral and ipsilateral-selective neurons, as well as competition between PS and sensory-evoked signals in L2/3. This demonstrates that recruitment of inhibition is the basis of competitive interactions between different cortical excitatory ensembles during awake behaving states^16,17,97^ and highlights the utility of combining patterned optogenetic stimulation and population calcium imaging to probe functional dynamics in neural circuits.^98–101^ PS responses were strikingly attenuated when paired with simultaneous whisker deflection. This suggests that strong inhibitory mechanisms are engaged during task performance to balance network excitation, which under our task conditions could accumulate across feedforward,^14,15,86,87,102^ feedback,^103^ lateral,^104,105^ and inter-hemispheric^91^ sources. In line with other recent studies, we also found that targeted stimulation of pyramidal neurons suppressed other non-targeted pyramidal neurons in the local L2/3 circuit.^37,98,105,106^ This provides further evidence that dense connectivity between excitatory and inhibitory neurons mediates strong lateral competition in cortex^10,88,105–107^ and that the recruitment of local inhibitory mechanisms scales with the number of concurrently activated pyramidal neurons.^37,97,104^

More generally, our results not only indicate that strong inhibitory pressure plays a major role in enforcing the sparsity of cortical responses,^16,88,97^ but that inhibition is selective. PS-evoked suppression of network neurons had the greatest proportional effects on responses that were initially small, consistent with previous findings indicating that disproportionate effects of cortical inhibition across heterogenous populations of barrel cortex neurons may serve to enhance sparse whisker coding.^104,108^ This suggests that interactions between excitatory and inhibitory microcircuits exhibit a specific structure to enhance the signal-to-noise ratio of robust sensory signaling while maintaining sparsity of responses.

### Small numbers of non-coding neurons can contribute to perception

Using all-optical interrogation, we demonstrate that targeted manipulation of surprisingly few L2/3 neurons can result in measurable biases in whisker-guided choices. This is consistent with recent barrel cortex studies showing that stimulation of small neural ensembles^37,109^ (and even single neurons^35,42^) can evoke perceptual effects. Surprisingly, we found that perceptual bias was significantly predicted by the number of non-coding neurons activated by targeted PS, challenging the consensus that decision-making processes read out activity from highly tuned stimulus-responsive neurons.^110^ Non-coding neurons did not show stimulus, movement, or reward-related activity, which is intriguing as recent studies have demonstrated that non-instructed movements can profoundly influence the activity of cortical neurons.^111,112^

In contrast with recent studies involving the selective optical stimulation of functionally tuned neurons during behavior,^43,45,46,100,109,113,114^ we did not find a significant effect of target ensemble tuning on task performance. One factor may be that in our experiments, target group whisker selectivity was relatively weak as we predominantly stimulated non-coding neurons (in particular because finding neurons with strong responses to both whisker input and 2P stimulation in the FOV was rare). In the future, this could be resolved by interrogating larger^99^ and/or volumetric FOVs^46,115^ to provide a larger sample of strongly tuned neurons. Thus, although further work is needed to investigate the differential contributions of contra- and ipsi-coding neurons to bilateral somatosensation, we provide compelling evidence that stimulus-non-coding neurons can be recruited to enhance sensory perception.

It remains to be established whether stimulus-non-coding neurons can influence performance in other perceptual contexts. Recent work in visual cortex suggests that stimulating non-coding neurons can even impair performance by suppressing the perceptually relevant cells in the local circuit,^45^ suggesting that the ability of non-coding neurons to influence perception may depend on task design or brain area. In our experiments, the dynamics of interhemispheric competition might engage a distinct decision-making process that prioritizes rapid decoding of intra-hemispheric spiking across a wider pool of neurons in barrel cortex in a winner-take-all scenario. Under perceptually ambiguous conditions, any additional intrahemispheric spikes may thus help reach a decision threshold, which could also reflect increased capacity for the photostimulated hemisphere to suppress the contralateral hemisphere,^84,85^ compensate for cortical adaptation to high-frequency whisker stimulation^116^ or enhance sensory responses in deeper cortical layers.^117,118^

### A reserve pool of silent neurons can be recruited to influence behavior

The observation of large fractions of stimulus-non-responsive neurons in cortex has long been intriguing.^23,47,48^ These “silent” neurons could be suppressed by intrinsic or synaptic mechanisms to constrain excessive cortical excitation, reduce coding redundancy, and/or increase energy efficiency via sparse coding.^50,119–121^ Our results suggest that under our task conditions, non-coding neurons are able to influence task performance but are normally suppressed by strong network inhibition during task-related whisker processing. Releasing these neurons from inhibitory suppression, as we achieved through patterned optogenetic stimulation but which could be achieved endogenously via disinhibitory VIP+ interneuron circuits^122^ and cortical plasticity,^22,49^ may therefore provide a simple mechanism for enhancing intracortical sensory signals in some contexts. Our findings also add causal support to emerging evidence showing that neurons in sensory cortex without stimulus-driven responses can contribute to neural processing.^123–127^ These results have important implications for designing novel therapeutic optical brain-machine interfaces and optogenetic therapies,^128^ since they suggest that it may not be necessary to precisely target optogenetic interventions to specific functionally defined pools of neurons. Rather, activation of a relatively small pool of pyramidal neurons regardless of functional identity could be sufficient to enhance sensory coding and restore some basic sensorimotor functions.

## Star★Methods

### Key Resources Table

**Table:** 

REAGENT or RESOURCE	SOURCE	IDENTIFIER
Bacterial and virus strains		
AAV1-hSyn-GCaMP6s-WPRE-SV40	UPenn Vector Core	AV-1-PV2824
AAVdj-CaMKIIa-C1V1(E162T)-TS-P2A-mCherry-WPRE	Stanford Virus Core	GVVC-AAV-048
AAV2/9-CaMKII-C1V1(t/t)-mScarlett-Kv2.1	Chettih and Harvey^98^	Gift from Harvey lab
AAV-DJ-EF1a-DIO-C1V1(E162T)-TS-p2A-mCherry	Stanford Virus Core	GVVC-AAV-50
Chemicals, peptides, and recombinant proteins		
Muscimol	Sigma-Aldrich	M1523-5MG
Experimental models: Organisms/strains		
Mouse: C57BL/6	Charles River	Strain code: 027
Mouse: PV-Cre; B6;129P2*-Pvalbtm1(cre)Arbr/*J	The Jackson Laboratory	Strain code: 008069
Software and algorithms		
Suite2p	https://github.com/cortex-lab/Suite2P	Pachitariu et al.^129^
Near automatic photoactivation response mapping ‘NAPARM’	https://github.com/llerussell/Naparm	Russell et al.^54^
PyBehaviour: behavioural control software	https://github.com/llerussell/PyBehaviour	Russell et al.^54^
PackIO	http://www.packio.org	Watson et al.^130^
DeepLabCut	https://github.com/DeepLabCut/DeepLabCut	Mathis et al.^131^
Matlab >2014a	MathWorks	https://www.mathworks.com

### Resource Availability

#### Lead contact

Further information and requests for resources and reagents should be directed to the lead contact, Michael Häusser (m.hausser@ucl.ac.uk).

#### Materials availability

This study did not generate new reagents or materials.

#### Data and code availability

- All data reported in this paper will be shared by the lead contact upon request
- This paper does not report original code
- Any additional information required to reanalyse the data reported in this paper is available from the lead contact upon request

### Experimental Model and Participant Details

All experimental procedures were carried out under license from the UK Home Office in accordance with the UK Animals (Scientific Procedures) Act (1986). Wild-type adult female mice (*Mus musculus*, C57BL/6, P35 - 42 on day of surgery) were used for all behavioral, imaging, and photostimulation experiments. PV-Cre mice (Jax 008069; P50 - 60 on day of surgery, n = 4 mice, 2 Male, 2 Female) were used for optogenetic photoinhibition experiments. Mice were maintained in a standard 12-hour light/dark cycle and single-housed in individually ventilated cages (IVCs) equipped with environmental enrichment to avoid whisker barbering when group-housed. Experiments and behavioral training usually occurred during the light cycle. Behavioral training usually commenced 1 week after surgery and took place for 2-3 weeks. Experiments were conducted for 2-3 weeks following task learning. To motivate task engagement, access to cage water was removed, and water (~1 ml per day, 5% sucrose solution) was received through daily behavioral training. Food was available *ad libitum* in the home cage. Body weight was maintained between 80%–90% for the duration of training/experiments. Daily health checks were performed to identify signs of poor health and excessive dehydration, and supplementary water was provided if necessary. Mice were not shared across different experiments and did not have any previous procedures.

### Method Details

#### Surgical procedures

Mice were implanted with a headplate, injected with virus and installed with a cranial imaging window in a single surgery session. Mice were first anesthetized with isoflurane (5% induction, 1.5% maintenance) and injected subcutaneously with an analgesic (Carprieve). The scalp was shaved with clippers then cleaned with iodine and a physiological *in vivo* external (IVE) solution (150 mM NaCl, 2.5 mM KCl, 10 mM HEPES, 2 mM CaCl_2_, 1 mM MgCl_2_) using sterile swabs (Sugi, Kettenbach). Mice were then fixed in a cranial stereotaxic frame and placed on a heat mat maintained at 37°C. Lidocaine was applied topically to the scalp before incision. Scalp was then removed bilaterally with surgical scissors revealing the dorsal surface of the skull. An aluminium headplate with a 7 mm diameter circular imaging well was fixed over the right hemisphere with dental cement (Super-Bond C&B, Sun-Medical).

A craniotomy (4 mm diameter) was made in the centre of the headplate well over S1 (2 mm anterior, 3.5 mm lateral from bregma) with a dental drill (NSK UK Ltd.), and the dura was removed using fine tweezers. Virus was front-loaded into a bevelled micropipette and injected at cortical depth of 300 μm at 0.1 μl/min (total volume 0.5 – 1 μl) using a calibrated oil-filled hydraulic injection system (Harvard apparatus). For one-photon optogenetic experiments, mice were co-injected with GCaMP6s (AAV1-hSyn-GCaMP6s-WPRE-SV40) and C1V1 (AAVdj-CaMKIIa-C1V1(E162T)-TS-P2A-mCherry-WPRE) in a 1:10 ratio. For two-photon imaging and targeted two-photon photostimulation experiments, mice were co-injected with GCaMP6s (AAV1-hSyn-GCaMP6s-WPRE-SV40) and somatically targeted C1V1 (st-C1V1; AAV2/9-CaMKII-C1V1(t/t)-mScarlett-Kv2.1). Stock st-C1V1 was diluted 1:10 in virus buffer, and GCaMP6s was added to the dilution mixture in a ratio of 1:10 GCaMP:st-C1V1. For PV-Cre mice, flexed-C1V1 (AAV-DJ-EF1a-DIO-C1V1(E162T)-TS-p2A-mCherry) was injected with GCaMP6s in a 1:10 GCaMP:C1V1 ratio. Opsin-negative control mice were injected with GCaMP6s diluted 1:10 in virus buffer. The needle was retracted slowly 5 minutes after the injection was completed and a cranial window (made from a 3 mm circular glass cover slip glued onto a 4 mm circular glass cover slip with optical glue NOR-61, Norland Optical Adhesive) was press-fit into the craniotomy and sealed in place with Vetbond and dental cement. Following completion of the surgery, mice were placed in an incubated recovery chamber and monitored closely until normal locomotor activity resumed. Post-operative care included daily weight and health monitoring, and Carprieve administration in the home cage water supply for 4-5 days.

#### Whisker stimulation

Contralateral and ipsilateral whiskers were deflected along the anterior-posterior axis with two piezoelectric actuators (Physik Instrumente; PL127.11) mounted on NOGA articulated arms (RS 785-7869) and positioned ~5 mm from the base of the whisker pad on either side of the snout. For single-whisker stimulation, the C2 whiskers were threaded into glass-capillaries glued to the piezo actuators and deflected in the anterior direction with a single 50 ms square pulse. For the single-whisker task every 1-2 weeks mice were briefly anesthetized to allow retrimming of the surrounding whiskers. Piezo deflection range was 0 - 1 mm from rest as calibrated with videography. For multi-whisker stimulation, whiskers were deflected with cardboard stimulus ‘paddles’ (2 x 3 cm). Multi-whisker stimulation consisted of a sinusoidal deflection for 500 ms at 20 Hz with uniform intensity. Stimulus intensity was modulated by varying the voltage signal amplitude used to drive the piezos. Analogue waveforms were generated using custom written MATLAB (MathWorks) scripts using the ’Data Acquisition Toolbox’, and outputted to single channel piezo drivers (Noliac, NDR6110) via a National Instruments card (USB-6351).

#### Behavioral training

We developed a bilateral decision-making task where head-fixed mice discriminated bilateral whisker input and reported decisions by licking left or right. Prior to training, mice were first habituated to experimenter handling and allowed to freely explore the head-fixation platform and tube. During head-fixation habituation, mice were trained to tolerate periods of head-restraint of increasing duration, and water rewards were delivered through the dual lickport to encourage pro-active directional licking.

We counterbalanced the spatial stimulus-response contingency across different mice. Mice were randomly assigned to either the symmetric (e.g., stimulus left, lick left) or asymmetric (e.g., stimulus left, lick right) task contingency and were not trained to switch between the two. To ensure reliable learning of the task framework, mice were initially trained on contra and ipsi unilateral stimuli (i.e., no distractor stimuli) of 100% intensity. Stimuli were delivered in a randomized order, with a maximum of 3 consecutive trials of the same type in a row. To promote associative learning of the stimulus-response contingency, during the first 2-3 sessions the target lick response was prompted with an automatic reward (‘autoreward’), which was unconditionally triggered 750 ms after the stimulus. Following 2-3 days of autoreward instruction, subsequent sessions had the autoreward feature disabled, requiring mice to respond correctly to obtain water rewards. Autoreward was occasionally and transiently re-enabled if mice displayed a strong response bias that persisted across multiple sessions. Correct trials were rewarded with a 5 μl water droplet (5 % sucrose solution), incorrect trials incurred a 5 s timeout while miss trials were not penalised. Mice were considered expert when performance on both stimulus-sides reached 70% on 3 consecutive days. For the single-whisker task, mice reached expert level within 7 sessions (days to reach expert: sym 6.1 ± 3.7; mean ± std; n = 31 mice, asym 6.8 ± 5.2; n = 30 mice; n.s. P > 0.05; Wilcoxon rank-sum test).

For the delayed-response task version, following whisker stimulation (0.5 s), mice withheld licking and were instructed to report their decision after a ~1.5 second delay (from stimulus onset) via an auditory go cue (piezo buzzer, 200 ms). To enforce this delay period, the lickports were mounted on a linear motor (P16 Mini Linear Actuator, Actuonix), which was driven via a linear actuator control board (LAC board, Actuonix). The lickports were moved in and out on each trial using a square driving signal generated in MATLAB (2 s duration, duty cycle 20%). The lickport motor took 170 ms to extend (a translation of 1 cm). Mice were trained in the delay task using the same shaping protocol described for the single-whisker non-delay task. During imaging and photostimulation experimental sessions, a post-hoc video-based measure of licking was derived to quantify early lick responses.

Following learning of the basic task framework, mice were transitioned to intensity discrimination training. Simultaneous bilateral deflections were delivered to the whiskers, and the difference in deflection intensity cued the target behavioral response in line with the learned task contingency. In discrimination sessions incorrect choices were not punished with negative reinforcement (beyond the omission of reward) to encourage mice to respond across all trial regardless of discrimination difficulty, and threshold stimulus trials were rewarded with 50% probability.

The behavioral task configuration was designed and controlled using PyBehavior (https://github.com/llerussell/PyBehavior). Behavioral training took place in custom built training boxes lined with sound attenuating foam. A dual lickport was constructed using syringe needles (manually filed down to a dull point) held in a plastic block (distance between syringe tips 5 mm), and a dual lick-o-meter circuit was used to detect licks electrically. Each lickport was gravity-fed with water through plastic tubing, which was gated by a solenoid pinch valve (225PNC1-11, NResearch). Reward volume was 5 μl (5 % sucrose solution). A set of USB computer speakers provided constant background white noise to mask sensory cues (e.g., piezo movement) and external noise. All behavioral training and experiments were performed in the dark.

#### Behavioral metrics

To track learning across sessions, we quantified the overall fraction of correct trial (P(Correct)) across contra and ipsi unilateral trials excluding miss trials. For quantification of discrimination accuracy, we analysed trials where mice reported a decision and excluded miss trials. For perceptual choices, we quantified the fraction of trials where mice reported the contra whisker ‘P(Report contra whisker)’. For motor (lickport) choices, we quantified the fraction of trials where mice licked the contra lickport ‘P(Lick contra lick-port)’. We fit psychometric data using the sigm_fit function in MATLAB (https://www.mathworks.com/matlabcentral/fileexchange/42641-sigm_fit). We used the fit to estimate the psychometric threshold stimulus (TS) as the corresponding stimulus condition that would result in 50% discrimination performance on the psychometric curve. For assessing the perceptual effect of optogenetic manipulation on TS trials discrimination, we quantified the difference in P(Report contra whisker) on control TS trials and opto+TS trials.

#### Behavioral videography

Behavioral videography was acquired during two-photon imaging and targeted two-photon photostimulation experiments using two infra-red (IR) sensitive CMOS cameras (DCC3240M Thorlabs) under infra-red LED illumination. One camera positioned face-on to the mouse (‘FaceCam’), recorded orofacial movements (licking and whisking) with a framerate of 75 – 100 fps. A second camera (‘Body-Cam’) recorded body movements at 20 fps. Acquisition parameters (FOV size, frame rate etc.) were configured using ThorCam (Thorlabs).

To analyse whisker pad and body movements, we used an ROI-based procedure to extract movement (in arbitrary units) in the video recordings. Rectangular ROIs were positioned over the contra and ipsi whisker pads and across the body. We computed the correlation coefficient ‘r’ between frame-to-frame pixel intensities as a measure of frame-to-frame similarity. Consecutive frames without any animal movement (e.g. no whisking) will have a ‘r’ close to 1. On the other hand, frames with a lot of movement will have a low ‘r’, as pixel intensities for a given pixel location will vary from moment to moment. Frame to frame whisking and body activity was therefore summarised as ‘1 – r’, normalised to the maximum value in the session.

We used DeepLabCut^131^ (DLC; https://github.com/DeepLabCut/DeepLabCut) to detect the tongue to quantify licking responses that occurred outside of the response window (whilst the motorised lickport was retracted). A single ‘global’ DLC model was trained using FaceCam video frames from across a sample of experiments. The model was trained using an equal proportion of licking / no licking frames (selected manually), with 10 ‘nodes’ manually labelled on the tongue (9 around the edge, 1 in the centre) on ~200 frames. On ‘test’ data, DLC tongue labels were assessed visually, and the model retrained if tracking accuracy was deemed poor. A binary video measure of licking (tongue present / not present) was calculated by assessing if 7 or more tongue labels were present in a given frame each with a ‘likelihood’ of ≥ 0.7.

#### Muscimol silencing

To allow pharmacological access to cortex, we installed cranial windows with a laser cut hole through the middle (500 μm diameter, Laser Micromachining, UK) plugged with silicone (Kwik-Sil) and centred over barrel cortex in 4 mice. The dura was removed before the cranial window was installed. Prior to the experiment, the silicone plug was removed from the laser-cut hole in cranial window, permitting access to cortex. Muscimol (powder dissolved in IVE; 100 nl at 5 μg/μl; Sigma-Aldrich) was manually pipetted onto the cranial window hole using a Gilson pipette and allowed to diffuse into barrel cortex for 20 minutes. Task performance was assessed before and after muscimol application, as well as 24 hours after application in a recovery session.

#### Widefield calcium imaging

Widefield GCaMP fluorescence imaging was performed to localize barrel cortex (and individual whisker barrels) through the cranial window 2-3 weeks following surgery (Figures S1A and S1C). Cortex was illuminated with a blue LED (Thorlabs) focused onto the cortical surface through a 4x/0.1-NA air objective (Olympus). GCaMP6s fluorescence was passed through a GFP excitation filter (Thorlabs) and detected using a CMOS camera (ORCA-Flash 4.0, Hamamatsu) via the objective (FOV ~1.5 x 1.5 mm). Contralateral whiskers were deflected at 10 Hz for 0.5 seconds during imaging. Stimulus-triggered average images were normalized to a 3 s pre-stimulus baseline window. Widefield imaging was performed before every imaging and photostimulation experiment to localize the 2P FOV. Imaging was performed at 10 fps, 15-20 stimulus repeats were delivered passively (i.e. not during task performance) to reduce licking/reward signal contamination.

#### One-photon optogenetics

One-photon photostimulation experiments were performed with an LED (595 nm, Thorlabs M595L3) mounted in the imaging light path above the two-photon objective. One-photon stimulation was delivered through the same objective as two-photon stimulation, positioned over the C2 whisker barrel in right hemisphere barrel cortex (localized using widefield imaging Figure S1A). LED power (mW) and triggering was controlled via an LED driver T-Cube (Thorlabs). Power was calibrated with a power meter (PM100A, Thorlabs). For the optogenetic substitution experiments presented in Figures 2A–2C (C1V1 in pyramidal neurons), the LED stimulus was a square pulse, 50 ms, power range: 0−50 mW. For photostimulation biasing experiments in Figure 2F (C1V1 in pyramidal neurons) the LED stimulus was a single square pulse (50 ms, 1 mW). For optogenetic photoinhibition experiments in Figure 2G (C1V1 expressed in PV-interneurons), the LED stimulus was a 500 ms continuous square pulse at 10 mW. Trials with optogenetic stimuli were not rewarded to avoid reinforcing behavioral responses and were interleaved with a high proportion of whisker trials to maintain task engagement. The only exception to this is the experiments presented in Figure S4D, where mice were intentionally trained to report optogenetic stimulation. A different cohort of mice was used for each experiment.

#### All-optical system design

We used an ‘all-optical’ experimental strategy^53–55,132^ to investigate the impact of sparse activation of L2/3 cortical ensembles on perception and local circuit physiology. All-optical experiments were performed using a customized dual beam path ‘all-optical’ microscope described previously^53^ A photostimulation light path coupled to a spatial light modulator (SLM) enabled spatially precise two-photon photostimulation (PS), while a second imaging light path provided optical readout of activity via 2P calcium imaging. Imaging data were acquired on a resonant scanning system (Bruker Corporation) at 30 Hz with 512 x 512-pixel resolution (FOV 470 x 470 μm, FOV depth: 150 – 200 μm; 256.5 ± 44.1 neurons per FOV). GCaMP6s was excited using a Chameleon Ultra II laser (Coherent) at 920 nm, which was raster scanned across the FOV. Static C1V1-mCherry and st-C1V1-mScarlet images were acquired at 765 nm. Imaging power under the objective was 50 mW. A 25x/0.95-NA water-immersion objective (Leica) was used for all experiments. Two-photon excitation of soma-targeted (st) C1V1 expressing neurons was performed with a femto-second pulsed laser at 1030 nm (Satsuma, Amplitude; 2 MHz repetition rate, average output 20 W, pulse width 280 fs) coupled to a spatial light modulator (SLM, 7.68 x 7.68 mm active area, 512 x 512 pixels, Meadowlark Optics/Boulder Nonlinear Systems). SLM phase masks were generated using the Gerchberg-Saxton algorithm^133^ and displayed on the SLM display using control software (Blink, Meadowlark). SLM targeting precision was ensured using calibration routines that mapped SLM pixel space onto 2P pixel space via an affine transformation.^53,54^ SLM-generated beamlets were simultaneously spiral scanned (three rotations, 15 ms duration per spiral, 15 μm diameter at 20 Hz for 500 ms) over somata with a pair of galvanometer mirrors. Photostimulation power was calibrated at 6 mW per cell. Power was modulated via an acoustic optical modulator (AOM), and calibrated with a power meter (PM100A, Thorlabs). Recordings were performed in 7 symmetric-trained mice (30 sessions, 4.3 ± 2.0 sessions per mouse) and 6 asymmetric-trained mice (22 sessions, 3.7 ± 1.8 sessions per mouse). During the targeted photostimulation experiment we delivered TS+PS (~15%), TS (~15%), PS (~15%), catch (~15%) and unilateral whisker trials (~40%) in a randomized order.

#### Photostimulation response mapping

Due to variable levels of GCaMP6s and st-C1V1 co-expression, not all cells in a FOV are addressable for all-optical interrogation. In order to identify cells with both functional opsin and indicator, we used a flexible photostimulation mapping procedure designed to enable rapid tests of the photostimulation responsivity of each cell in the FOV (Near automatic photoactivation response mapping: ‘*NAPARM*’^54^). Static expression images of GCaMP and st-C1V1 were loaded into NAPARM’s user GUI, and 350 pixel targets (corresponding to the xy position of cell somata in the 2P FOV) were selected semi-automatically based on local intensity maxima. The 350 photostimulation target sites were clustered into 7 target groups of 50 cells using ek- means, and a phase-mask and a set of photostimulation galvanometer position coordinates for each cluster was generated. Sequential photostimulation of each target group was performed by the photostimulation module of the all-optical system and the SLM software and was triggered using synchronisation software (PackIO; https://github.com/apacker83/PackIO). 15 photostimulation repeats of the stimulation sequence (stim pattern 1 through to 7) were performed (total of 105 photostimulation events per experiment). Photostimulation parameters were 15 ms spiral, 15 μm diameter, 20 Hz, 500 ms duration, 10 reps, 5 s ITI. Photostimulation responsive neurons were identified by comparing baseline ∆F/F responses (mean in a 1 s pre-stimulus window) to evoked ∆F/F responses (mean of 0.5 to 1 s post-stimulus) with a one-tailed Wilcoxon ranked-sum test (P < 0.05 = responsive cell).

#### Two-photon calcium imaging data

All two-photon imaging data were motion-corrected and segmented into somatic and neuropil fluorescence traces using the Python release of Suite2p^129^ (https://github.com/MouseLand/suite2p). Manual curation was performed to discard ROIs with non-somatic shapes (e.g. dendritic/axonal processes) or filled nuclear fluorescence. Neuropil subtraction was performed across cells by subtracting the neuropil signal from the somatic signal. Prior to subtraction, the neuropil signal was scaled by a coefficient (ranging from 0 – 1), which was based on an estimation of neuropil signal contamination in the somatic signal using robust linear regression.^134^

For each neuron and each trial, the neuropil-subtracted fluorescence signal was extracted 1 second pre-stimulus to 6 seconds post-stimulus (210 frames in total at 30 fps). Each trial trace was normalised using a ∆F/F calculation, where F was the average fluorescence signal across the 1 second pre-stim baseline (30 imaging frames). The evoked response on a given trial was subsequently calculated as the average ∆F/F signal 0.5–1 second post-stimulus (15 imaging frames). This analysis window avoids both sensory and photostimulus presentation (which last 0.5 s) and avoids licking. Thus, the imaging response window is optimized to measure the stimulus-evoked signal prior to contamination from response/reward related activity. During behavioural imaging sessions we confirmed this analysis window was free from licking using videography analysis (average video-detected RT post-stimulus = 1662.4 ± 142 ms; mean ± std; n = 52 sessions; Figures S6A and S6B). Any trials with licking detected during the analysis window were not included in analyses.

#### Identifying neurons with significant trial responses

Neurons with significant trial-evoked responses were identified by comparing the evoked ∆F/F response (during the delay epoch 500–1,000 ms after stimulus onset) distributions across different trial conditions using a one-tailed Wilcoxon rank-sum test and a P < 0.05 threshold. For example, neurons activated by photostimulation (PS) were identified by comparing PS trial to Catch trial response distributions. Likewise, neurons activated by photostimulation on top of sensory input (TS+PS) were identified by comparing responses on TS trials to TS+PS trials. Neurons significantly selective for contra vs ipsi whisker stimulation were defined by comparing trial-evoked responses on contra and ipsi trials.

#### Pixel-wise analysis of imaging data

Pixel-wise analysis was performed on the raw calcium imaging data to corroborate trace-based analyses. This was important for confirming that Suite2p-detected responses (e.g. stimulus-selective / photostimulation activated) reliably reflected measurable and visible changes in somatic fluorescence (as opposed to neuropil contamination or passing axonal/dendritic processes). For each trial, registered imaging frames were normalised (∆F/F, as described above) to a 1 second pre- stimulus baseline, and STA response was assessed as the average response (500–1,000 ms) post stimulus. Pixelwise STAs ‘stamps’ for individual cells were generated by sampling a mini-FOV 50 x 50 μm with each cell centred in the stamp at pixel location (25,25).

#### Receiver operating characteristic analysis

We used ROC analysis (MATLAB’s perfcurve function) to assign a stimulus-selectivity score to each neuron according to how well an ideal observer could decode trial-type (contra vs. ipsi) from the evoked response. Stimulus selectivity was defined as the area under the ROC performance curve (AUC). Neurons with stimulus selectivity > 0.5 were selective for contra trials, and neurons < 0.5 were selective for ipsi trials. We used the same procedure to quantify each neuron’s correlation with behavioral choices on threshold trials by calculating action probability (comparison of 21.7 ± 15.7 ‘lick contra lickport’ and 24 ± 14.9 ‘lick ipsi lickport’ trials), choice probability (comparison of 21.6 ± 14.6 ‘report contra whisker’ and 24.1 ± 15.3 ‘report ipsi whisker’ trials) and detect probability (comparing lick vs no lick trials). Choice probability scores > 0.5 indicate increased fluorescence on trials with contra lickport choices for symmetric-trained mice, but ipsi lickport choices for asymmetric-trained mice. To calculate whisker responsiveness scores we compared TS and catch trial responses, and adjusted this AUC metric between -1 and 1 such that the sign denoted whether a neuron was positively or negatively modulated by TS input.

#### Control trial resampling

To test whether the relationship between photostimulation target count and perceptual bias reflected an underlying correlation between the number of active neurons and perceptual choice we performed a shuffle test based on resampling non-photostimulation trials (as shown in Figure S11B). For each experiment, we sub-sampled (with re-sampling) control TS trials, to match the corresponding number of photostimulation trials. This was possible as TS trials were delivered with twice the frequency compared to each of the TS+PS trial conditions. We then compared the choice deviation ‘∆P(Report contra whisker)’ of this sub-sample to the average of the control TS trials, and summed the number of photostimulation target neurons that were identified as being significantly activated. We did this twice for each experiment to generate a ‘fake’ TS+PS dataset, and then assessed the correlation between choice bias and photostimulation target activity. We repeated this procedure 10000 times to build up a distribution of correlation coefficients against which we could compare the true photostimulated trials.

## Quantification and Statistical Analysis

### Statistical analyses

Unless otherwise stated all paired statistical comparison tests used were Wilcoxon signed-rank tests. For unpaired comparisons Wilcoxon rank-sum tests were used. We did not use methods to test for normality of sample distributions. A linear regression model (Matlab ‘*fitlm*’) was used to predict photostimulation-evoked choice bias using various photostimulation and behavioral parameters. All inputs to the linear regression model were first z-scored. Scatter plots show least-squares lines, and Pearson’s correlation coefficients were calculated using the Matlab function “*corrcoef*.” NS, not significant. * P < 0.05, ** P<0.01, *** P<0.001. All data are reported as the mean and standard error of the mean (SEM) across individual experimental sessions (unless otherwise stated). Details of statistical tests and sample sizes can be found in the results text and in figure legends. No statistical methods were used to predetermine sample size. Experimenters were not blind to experimental conditions.

## Supplementary Material

Supplementary Information

## Highlights

- All-optical interrogation of barrel cortex during bilateral whisker discrimination
- Sparse coding of contralateral and ipsilateral whisker information
- Selective sensory-evoked inhibition helps ensure sparse coding
- Optogenetic recruitment of stimulus non-coding neurons can aid perception

## In brief

Gauld et al. use all-optical interrogation to probe sparse coding in the barrel cortex. They find that targeted photostimulation of task-silent neurons evokes perceptual choice bias and that these neurons are selectively suppressed by inhibition during whisker processing. Their findings show that silent neurons can be recruited to enhance behavior.
